# *Moringa concanensis* L. Alleviates DNCB-Induced Atopic Dermatitis-like Symptoms by Inhibiting NLRP3 Inflammasome-Mediated IL-1β in BALB/c Mice

**DOI:** 10.3390/ph15101217

**Published:** 2022-09-30

**Authors:** Kyeong-Min Kim, So-Yeon Kim, Tamanna Jahan Mony, Ho Jung Bae, Seung-Hyuk Choi, Yu-Yeong Choi, Ju-Yeon An, Hyun-Jeong Kim, Ye Eun Cho, Kandhasamy Sowndhararajan, Se Jin Park

**Affiliations:** 1 Department of Food Biotechnology and Environmental Science, Kangwon National University, Chuncheon 24341, Korea; 2 Agriculture and Life Science Research Institute, Kangwon National University, Chuncheon 24341, Korea; 3 Department of Botany, Kongunadu Arts and Science College, Coimbatore 641029, India; 4 School of Natural Resources and Environmental Sciences, Kangwon National University, Chuncheon 24341, Korea

**Keywords:** atopic dermatitis, keratinocyte, IL-1β, NLRP3 inflammasome, *Moringa concanensis*

## Abstract

Atopic dermatitis (AD) is a chronic inflammatory skin disease characterized by pruritus, dry skin and redness on the face and inside elbows or knees. Most patients with AD are children and youths, but it can also develop in adults. In the therapeutic aspect, treatment with corticosteroids for AD has several side effects, such as weight loss, atrophy and acne. In the current study, we examined the anti-inflammatory effect of *Moringa concanensis* leaves on HaCaT keratinocytes and 2,4-dinitrochlorobenzene (DNCB)-induced atopic dermatitis-like symptoms in BALB/c mice. We observed that *M. concanensis* treatment exhibited significant inhibition in the production of inflammatory mediators and proinflammatory cytokines, such as IL-1β, in LPS-induced HaCaT keratinocytes by downregulating the NLRP3 inflammasome activation. Moreover, *M. concanensis* inhibited the activation of JNK, AP-1 and p65, which resulted in the deformation of NLRP3 in LPS-stimulated HaCaT cells. In mice with DNCB-induced AD-like skin lesions, the administration of *M. concanensis* ameliorated the clinical symptoms, such as the dermatitis score, thickness of lesional ear skin and TEWL. Furthermore, *M. concanensis* could attenuate the activation of the immune system, such as reducing the spleen index, concentration of the IgE levels and expression of the NLRP3 inflammasome in ear tissues. Therefore, our results suggest that *M. concanensis* exerts anti-atopic dermatitis effects by inhibiting the NLRP3 inflammasome-mediated IL-1β.

## 1. Introduction

From various pathophysiological perspectives, atopic dermatitis (AD) is a chronic relapsing inflammatory skin disease associated with pruritus and redness, typically on the face and the inside of elbows and knees [1]. Therefore, AD is closely connected to patients’ quality of life and comorbidities [2]. AD can occur at any age and its prevalence is increasing. According to one report, the number of AD patients diagnosed in one year ranged from 13.5% to 41.9%, depending on the country [3]. The main symptoms of AD are characterized by a multidimensional patient burden, including persistent itching, dryness of the skin and depression [4,5]. Although the pathogenesis of AD is still unclear, some studies have suggested that most patients with AD are affected due to sensitization to environmental allergens, genetic backgrounds and increased serum immunoglobulin E (IgE) [6,7]. Based on clinical experiments, topical corticosteroids are commonly used in patients with AD [8]. However, the current treatments for AD, especially topical steroids, have various adverse effects, such as atrophy, acne and red burning skin [9]. Therefore, it is necessary to develop both effective and safer therapies for atopic dermatitis without side effects.

The skin barrier is an important defense system against potential allergens, microorganisms and pollutants [10]. When the barrier of the epidermis is damaged, there is increased epicutaneous absorption of environmental allergens and the activation of the immune response, which could stimulate the expression of inflammatory cytokines such as IL-4, IL-33 and IL-1β [11]. Several studies have reported that the nucleotide-binding oligomerization domain, leucine-rich repeat and pyrin domain-containing 3 (NLRP3) inflammasome, which includes NLRP3, ASC and caspase-1, can regulate the activation of IL-1β [12,13]. NLRP3 inflammasome-dependent IL-1β activation by cleaved caspase-1 is the main inflammatory cytokine in the progression of inflammation [14]. It was thought that the inflammasome could only be expressed in immune cells, but recent studies suggest that human keratinocytes result in the expression of IL-1β by activating the NLRP3 inflammasome [15]. 

*Moringa concanensis* Nimmo (Morigaceae) is an medicinally important plant, which is called Kattumurungai or Peyimurungai in Tamil [16]. *M. concanensis* inhabits India and Asian and Arab countries. It has been reported that *M. concanensis* leaves can be used to treat different medical conditions, including dysmenorrhea, hypertension, constipation and skin tumors [17]. Moreover, it has been reported that *M. concanensis* contains flavonoid compounds, such as quercetin [18]. Although the beneficial properties of *Moringa oleifera* in atopic dermatitis have been widely reported [19,20], the effects of *M. concanensis* in atopic dermatitis are still unknown. In this study, we investigated the anti-inflammatory and the therapeutic effects of *M. concanensis* leaves in LPS-stimulated HaCaT keratinocytes and a 2,4-dinitrochlorobenzene (DNCB)-induced atopic dermatitis murine model.

## 2. Results

### 2.1. Analysis of Quadrupole Time-of-Flight (Q-TOF) Mass Spectrometry of M. concanensis

**Figure 1:**
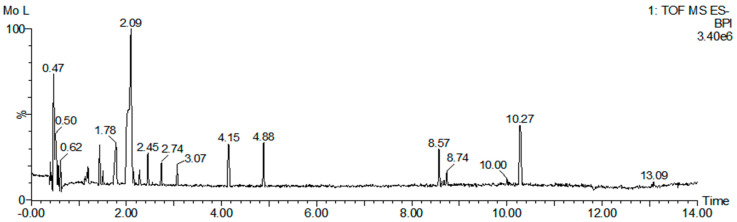
UPLC−QTOF−MS/MS analysis of *M. concanensis* L. Base peak intensity (BPI) chromatogram.

To identify the major phytochemicals in the ethanol extracts of *M. concanensis* leaves, we conducted a QTOF-MS/MS analysis. The results of the QTOF-MS/MS analysis revealed the presence of 313 phytochemicals in the leaves of *M. concanensis* (Figure 1 and Table 1). The major phytochemicals included quinic acid (C_7_H_12_O_6_), coumaroylquinic acid (C_16_H_18_O_8_), coumaric acid (C_9_H_8_O_3_) and quercetin (C_15_H_10_O_7_). It has been indicated that quinic acid derivatives, coumaric acid and quercetin suppress immune responses [21,22,23]. In particular, some studies revealed that quercetin downregulated the production of IL-1β by inhibiting the NLRP3 inflammasome [24,25]. 

### 2.2. M. concanensis Inhibits the LPS-Stimulated Inflammatory Mediators in HaCaT Cells 

**Figure 2:**
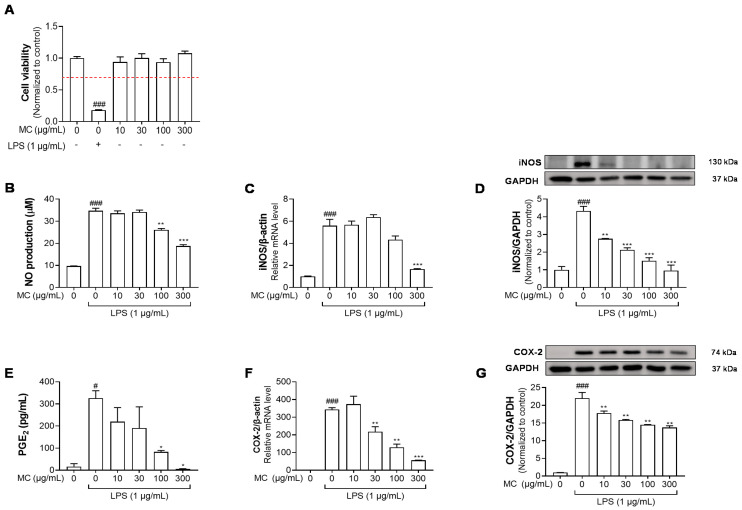
Effects of *M. concanensis* on the inflammatory response of LPS−stimulated HaCaT cells. HaCaT cells were treated with *M. concanensis* (MC) for 24 h, and cell viability was determined by MTT assay (**A**). Cells were pretreated with MC for 1 h before LPS stimulation (1 μg/mL) for 24 h. The production of NO and PGE_2_ was determined by Griess reagent and ELISA kits, respectively (**B**,**E**). The level of mRNA expressions of iNOS and COX-2 was determined by RT-qPCR (**C**,**F**). The levels of iNOS and COX-2 proteins were measured by Western blotting analysis, and the quantifications were normalized to the control (**D**,**G**). The data presented are mean of three independent determinations and indicate the mean ± S.E.M. ^#^ *p* < 0.05, ^###^ *p* < 0.001 compared to the vehicle-treated controls; * *p* < 0.05, ** *p* < 0.01 and *** *p* < 0.001 compared to the LPS-treated group.

Recently, new insight into the pathogenesis of AD focused on abnormalities in the epidermal layer [26]. Furthermore, several studies have suggested that the downregulation of immune activation in epidermal keratinocytes plays a key role in ameliorating inflammatory skin diseases, such as AD [27,28]. Therefore, we investigated the effect of *M. concanensis* on LPS-stimulated inflammatory responses in keratinocytes. Firstly, an MTT assay was performed to determine the cytotoxic concentration of *M. concanensis* in HaCaT keratinocytes. The treatment of *M. concanensis* extract did not affect the viability of HaCaT cells at the concentration of 10–300 μg/mL (Figure 2A). HaCaT cells were pretreated with *M. concanensis* for 1 h and then treated with LPS (1 μg/mL) for 24 h. *M. concanensis* extract markedly reduced the production of NO and PGE_2_ at 100 and 300 μg/mL concentrations (Figure 2B,E). Moreover, *M. concanensis* inhibited the mRNA as well as protein expressions of iNOS and COX-2, which is related to the synthesis of NO and PGE_2_ production (Figure 2C,D,F,G). Our results suggest that *M. concanensis* can regulate the production of inflammatory mediators, such as NO and PGE_2_, via the inhibition of iNOS and COX-2 expressions.

### 2.3. M. concanensis Downregulated the Expression of Inflammatory Cytokines in LPS-Stimulated HaCaT Cells

**Figure 3:**
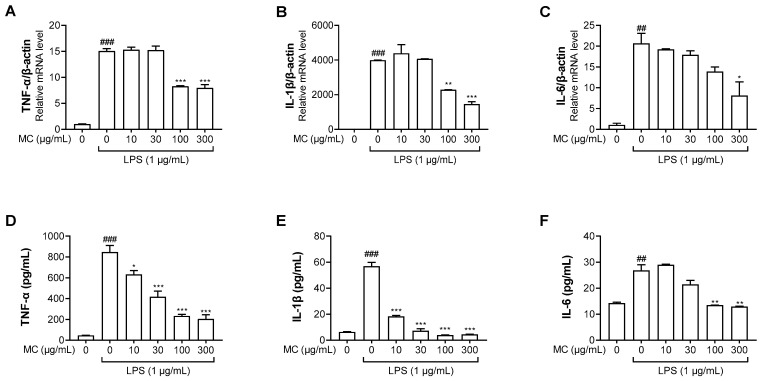
Effects of *M. concanensis* on LPS−stimulated proinflammatory cytokine expression in HaCaT keratinocytes. Cells were pretreated with *M. concanensis* (MC) for 1 h before LPS stimulation (1 μg/mL) for 24 h. The level of mRNA expressions of TNF-α, IL-1β and IL-6 was determined by RT-qPCR (**A**–**C**). The level of TNF-α, IL-1β and IL-6 proteins was measured by ELISA kits (**D**–**F**). The data presented are the mean of three independent determinations and indicate the mean ± S.E.M. ^##^ *p* < 0.01, ^###^ *p* < 0.001 compared to the vehicle-treated controls; * *p* < 0.05, ** *p* < 0.01 and *** *p* < 0.001 compared to the LPS-treated group.

The production of inflammatory cytokines, such as TNF-α, IL-1β and IL-6, due to the inflammatory reactions could control immune activation [29]. Therefore, we examined whether *M. concanensis* had an inhibitory effect on inflammatory cytokines, such as TNF-α, IL-1β and IL-6, in LPS-stimulated HaCaT cells. The cells were pretreated with *M. concanensis* for 1 h before LPS stimulation (1 μg/mL) for 24 h. The LPS-stimulated cells and mediums were collected to investigate the expression of inflammatory cytokines using RT-qPCR and ELISA kits. Figure 3A–C show that *M. concanensis* treatment significantly reduced the mRNA expression levels of TNF-α, IL-1β and IL-6 compared to those in the LPS-treated controls in HaCaT cells. Moreover, the secretion of TNF-α, IL-1β and IL-6 protein was markedly reduced by *M. concanensis* (Figure 3D–F).

### 2.4. M. concanensis Reduced the Expression of IL-1β by Inhibiting the NLRP3 Inflammasome in HaCaT Cells

**Figure 4:**
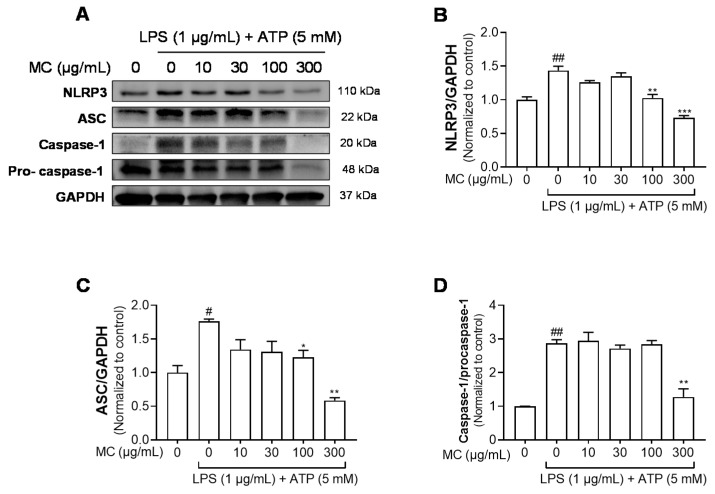
Effects of *M. concanensis* on the NLRP3 inflammasome activation in HaCaT keratinocytes. Cells were pretreated with *M. concanensis* (MC) for 1 h before LPS stimulation (1 μg/mL) for 24 h and ATP (5 mM) for 1 h. The protein expressions of NLRP3, ASC and Caspase-1 in HaCaT cells were determined by immunoblot analysis (**A**–**D**). The data presented are the mean of three independent determinations and indicate the mean ± S.E.M. ^#^ *p* < 0.05 and ^##^ *p* < 0.01 compared to the vehicle-treated controls; * *p* < 0.05, ** *p* < 0.01 and *** *p* < 0.001 compared to the LPS-treated group.

The NLRP3 inflammasome is a multiprotein complex that consists of NLRP3, ASC and caspase-1. The NLRP3 inflammasome initiates immune responses during exposure to a variety of stimuli, mainly pathogen and danger-related molecular patterns [30]. The activation of the inflammasome results in the secretion of cytokine IL-1β, which is correlated with chronic inflammatory diseases [31]. As shown in Figure 3B,E, we found that *M. concanensis* effectively reduced the expression of IL-1β in HaCaT cells stimulated with LPS. Therefore, we next examined the effects of *M. concanensis* on the NLRP3 inflammasome activation in LPS/ATP-stimulated HaCaT cells. The experimental data demonstrated that *M. concanensis* dose-dependently downregulated the expression of NLRP3 (Figure 4B). Furthermore, *M. concanensis* significantly attenuated the activation of ASC and cleaved caspase-1 at 300 μg/mL (Figure 4C,D). These data indicate that *M. concanensis* reduced the secretion of IL-1β by regulating the formation of the NLRP3 inflammasome.

### 2.5. M. concanensis Inhibited the Phosphorylation of NF-κB, MAPK and AP-1 in HaCaT Cells Stimulated with LPS

**Figure 5:**
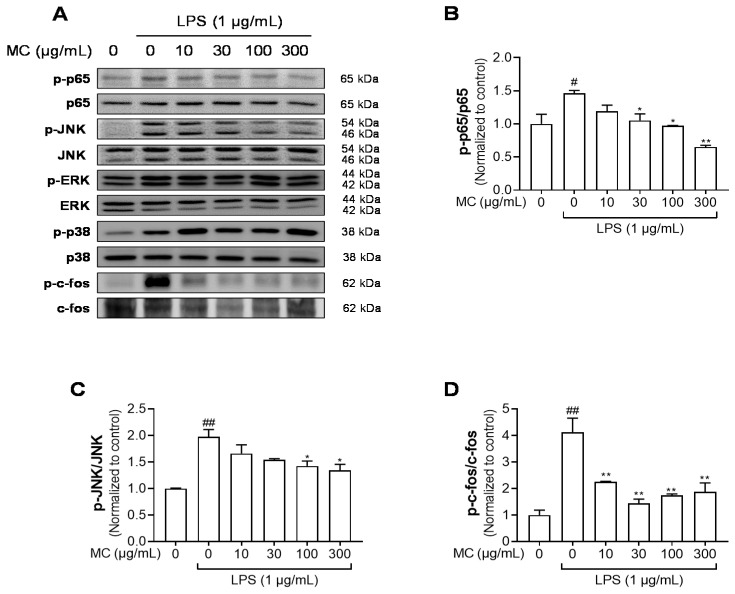
Effects of *M. concanensis* on MAPK/AP-1/NF-κB signaling in HaCaT keratinocytes. Cells were pretreated with *M. concanensis* (MC) for 1 h prior to LPS stimulation (1 μg/mL) for 1 h. The expression of phospho-p65, JNK, p38, ERK, c-fos, p65, JNK, p38, ERK and c-fos was measured by a Western blot analysis (**A**). The phosphorylation level was normalized to the control (**B**–**D**). The data presented are the mean of three independent determinations and indicate the mean ± S.E.M. ^#^ *p* < 0.05 and ^##^ *p* < 0.01 compared to the vehicle-treated controls; * *p* < 0.05 and ** *p* < 0.01 compared to the LPS-treated group.

ROS and NF-κB contribute to the mechanism underlying NLRP3 inflammasome activation [32]. Mounting evidence indicates that activated transcription factors, such as NF-κB, induce the priming of the NLRP3 inflammasome [33,34]. Hence, we studied whether *M. concanensis* suppressed the phosphorylation of NF-κB and its upstream MAPK in LPS-stimulated HaCaT cells. As shown in Figure 5A,B, LPS treatment significantly upregulated the phosphorylation of NF-κB. However, *M. concanensis* treatment significantly downregulated the phosphorylation of p65. Furthermore, LPS treatment increased the phosphorylation of JNK, p38 and ERK which are subunits of MAPK, but *M. concanensis* only exhibited a significant reduction in the phosphorylation of JNK (Figure 5A,C). In addition, AP-1, which is regulated by the activated MAPK family, such as JNK, can mediate the transcription of inflammatory mediators [35]. Our results show that *M. concanensis* also inhibited the phosphorylation of the AP-1 subunit c-fos (Figure 5A,D). Therefore, these results demonstrate that *M. concanensis* had anti-inflammatory properties and inhibited the priming of the NLRP3 inflammasome via the inhibition of phosphorylated p65, JNK and c-fos signaling.

### 2.6. M. concanensis Improved the Clinical Symptoms in Mice with AD-like Skin Lesions Induced by DNCB

**Figure 6:**
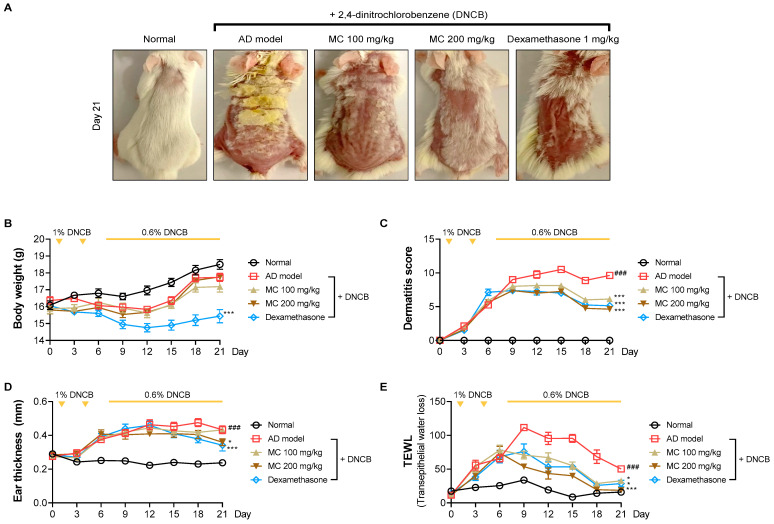
Effects of *M. concanensis* on atopic dermatitis in BALB/c mice induced by DNCB. The dorsal skin images were obtained at identical magnification, and representative images obtained on day 21 are shown (**A**). The clinical symptoms, including body weight (**B**), dermatitis score (**C**), ear thickness (**D**) and TEWL (**E**), were measured every 3 days (n = 8). The data presented are the mean ± S.E.M. ^###^ *p* < 0.001 versus Normal. * *p* < 0.05 and *** *p* < 0.001 compared to the DNCB-treated group. AD model; only DNCB-treated group, MC 100 mg/kg + DNCB; DNCB-induced mice administered with 100 mg/kg of *M. concanensis*, MC 200 mg/kg + DNCB; DNCB-induced mice administered with 200 mg/kg of *M. concanensis*.

Given the anti-inflammatory properties of *M. concanensis* in keratinocytes, we further investigated whether *M. concanensis* has anti-atopic dermatitis effects in mice with AD-like skin lesions induced by DNCB. BALB/c mice were shaved using a clipper for dorsal skin. After shaving, the mice were sensitized with 1% DNCB twice every 7 days. To evaluate the effects of *M. concanensis* on AD, the mice were orally administered with *M. concanensis* (100 and 200 mg/kg) for 14 days. In addition, 0.6% DNCB was topically applied to accelerate atopic dermatitis once every 2 days. There was no difference in the body weight of the AD mice in the *M. concanensis*-treated group, but a significant reduction in the body weight of dexamethasone (1 mg/kg)-administered group when compared with the DNCB-treated group (Figure 6B). A significant increase in the SCORAD index was observed in the DNCB-treated group compared with the normal group. However, we observed that the dermatitis scores were dose-dependently reduced by the administration of *M. concanensis* when compared with the DNCB-treated group (Figure 6A,C). Moreover, the mice treated with *M. concanensis* had reduced ear thickness and TEWL compared to the DNCB-treated group (Figure 6D,E). These results demonstrate that the *M. concanensis* oral administration can attenuate the clinical symptoms of AD without side effects, such as body weight loss, in a DNCB-induced AD mice model.

### 2.7. M. concanensis Ameliorated the Immunological and Histological Changes in DNCB-Challenged BALB/c Mice

**Figure 7:**
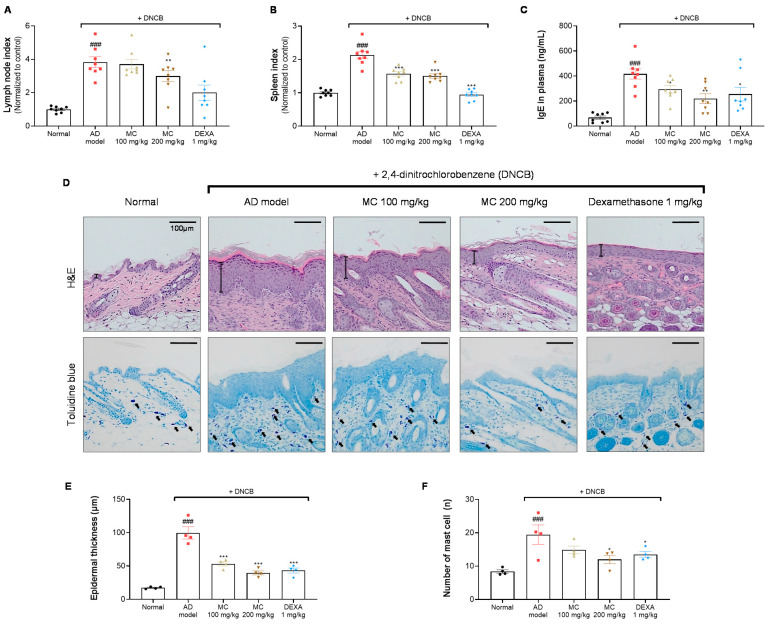
Effects of *M. concanensis* on histological and immunological changes in DNCB-induced AD. On day 21, the mice were sacrificed to investigate the immunological and histological differences. The lymph node (**A**) and spleen (**B**) indices were normalized to those in the normal group [Index = (organ weight/body weight) × 100] (n = 8). The plasma IgE concentration (**C**) was measured by an ELISA kit (n = 8). Images of hematoxylin and eosin (H&E) and toluidine blue (TB) staining of lesional dorsal skin (**D**) were obtained at 200× magnification, and characteristic images are shown; scale bar: 100 μm. Epidermal thickness (**E**) and the number of mast cells (**F**) were counted (n = 4), and the data presented are mean ± S.E.M. ^###^ *p* < 0.001 versus Normal. * *p* < 0.05, ** *p* < 0.01 and *** *p* < 0.001 versus the only DNCB-treated group. AD model; only DNCB-treated group, MC 100 mg/kg + DNCB; DNCB-induced mice administered with 100 mg/kg of *M. concanensis*, MC 200 mg/kg + DNCB; DNCB-induced mice administered with 200 mg/kg of *M. concanensis*, DEXA 1 mg/kg + DNCB; DNCB-induced mice administered with 1 mg/kg of dexamethasone.

To determine whether the administration of *M. concanensis* affects immunological activation, the weights of the cernical lymph nodes and spleen and the plasma IgE concentration were calculated after sacrifice. The indices of the lymph nodes and spleen were significantly increased in the DNCB-only group when compared with the normal group. Although the lymph node index did not significantly decrease, the spleen index was significantly reduced by the oral administration of *M. concanensis* when compared with the DNCB-administered group (Figure 7A,B). In addition, it is well known that the upregulated IgE levels have been detected in patients with AD [36]. As shown in Figure 7C, *M. concanensis* dose-dependently inhibited the level of the plasma IgE compared to that in the DNCB-only group. These data indicate that the reduced spleen index and IgE levels could be therapeutic strategies in AD therapies.

To investigate the histological changes, epidermal hyperplasia and mast cell infiltration in lesional dorsal skin were investigated by H&E and toluidine blue staining, respectively (Figure 7D). A significant increase in the epidermal thickness in the DNCB-administered group was observed when compared with the normal group (Figure 7D,E). Furthermore, mast cell infiltration was considerably increased in the DNCB-challenged group (Figure 7D,F). However, the mice treated with *M. concanensis* showed a dose-dependent suppression of hyperplasia and mast cell infiltration in lesional dorsal skin tissues (Figure 7D,E,F). These data suggest that *M. concanensis* may regulate of the immune system activation and histological changes in DNCB-induced lesional dorsal skin.

### 2.8. M. concanensis Inhibited the Activation of the NLRP3 Inflammasome in DNCB-Treated BALB/c Mice

**Figure 8:**
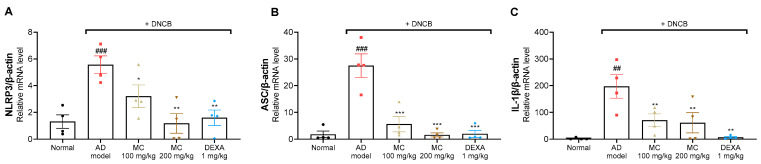
Effects of *M. concanensis* on the NLRP3 inflammasome in DNCB-induced AD lesional ear tissues. The mRNA levels of NLRP3 (**A**), ASC (**B**) and IL-1β (**C**) in DNCB-induced lesional ear tissue were measured by RT-qPCR (n = 4). The data presented are the mean of three independent determinations and indicate the mean ± S.E.M. ^##^ *p* < 0.01 and ^###^ *p* < 0.001 versus Normal. * *p* < 0.05, ** *p* < 0.01 and *** *p* < 0.001 versus the only DNCB-treated group. AD model; only DNCB-treated group, MC 100 mg/kg + DNCB; DNCB-induced mice administered with 100 mg/kg of *M. concanensis*, MC 200 mg/kg + DNCB; DNCB-induced mice administered with 200 mg/kg of *M. concanensis*, DEXA 1 mg/kg + DNCB; DNCB-induced mice administered with 1 mg/kg of dexamethasone.

It has been shown that the upregulation of NLRP3 inflammasome is associated with the pathogenesis of chronic dermatitis in the skin of mice [37]. To determine whether the symptoms of AD were attenuated via the inhibition of the NLRP3 inflammasome, we investigated whether the application of *M. concanensis* inhibited the NLRP3 inflammasome in DNCB-induced lesional ear tissues. After the sacrifice, the lesional ear tissues were collected and analyzed for the expression of NLRP3, ASC and IL-1β. As shown in Figure 8, the NLRP3, ASC and IL-1β expressions were significantly increased in the DNCB-treated group. However, similar to Figure 4, we found that the mice treated with *M. concanensis* had significantly reduced NLRP3, ASC and IL-1β mRNA expression compared to mice in the DNCB-treated group (Figure 8A–C). Therefore, the results of the present study suggest that *M. concanensis* relieved AD-like symptoms by downregulating the expression of NLRP3, IL-1β and ASC in DNCB-induced lesional ear tissues.

## 3. Discussion

Atopic dermatitis is known as a typical chronic inflammatory disease, and its prevalence in patients with AD has consistently increased over the last decade [38]. This inflammatory skin disease is normally demonstrated during the first year of birth, however, it can occur in adults [39]. The pathogenesis of AD involves complex factors, including environmental provocation, genetic predisposition and immunological abnormalities [40]. Based on clinical research, AD cannot be completely cured [41]. Therefore, the main management of AD involves improving the clinical symptoms and achieving long-term disease control following treatment guidelines. The drugs used in the treatment of AD, such as glucocorticosteroids, antihistamines and calcineurin inhibitors, can improve itching, edema and skin inflammation [42]. However, several studies have shown that the prolonged use of these medications could cause various adverse effects, including skin atrophy, heart failure and high blood pressure [43,44]. In this study, we first found that *M. concanensis* alleviated AD-like lesions in BALB/c mice induced by DNCB. Furthermore, we revealed that *M. concanensis* blocked NLRP3 formation by inhibiting the JNK-NF-κB and AP-1 pathways. These observations indicate that *M. concanensis* could be a novel candidate for preventing and treating AD.

Keratinocytes play a potential role in skin immune responses that cause immune cells to produce proinflammatory cytokines [45]. The present study exhibited the anti-inflammatory properties of *M. concanensis* and its underlying mechanisms in LPS-stimulated HaCaT keratinocytes. After Toll-like receptor 4 (TLR4) recognizes LPS, the TLR4 signaling cascade regulates inflammatory mediators via the phosphorylation of transcription factors, mainly NF-κB [46,47]. A recent study indicated that the NF-κB activation could positively regulate the NLRP3 inflammasome, which aggravated immune-related skin diseases, such as AD [48]. In LPS-induced HaCaT keratinocytes, it was observed that *M. concanensis* reduced iNOS and COX-2 expressions, which synthesize NO and PGE_2_, inhibiting the secretion of inflammatory mediators. Furthermore, we found that *M. concanensis* inhibited the level of mRNA and protein expressions of TNF-α, IL-1β and IL-6 by inhibiting the phosphorylation of JNK/AP-1/NF-κB. Moreover, we confirmed that *M. concanensis* reduced the expression of IL-1β by inhibiting the formation of the NLRP3 inflammasome. The genus *Moringa* is known as a medicinal plant that has been traditionally used for diseases such as colds and diabetes [49]. Many *Moringa* species have been reported to inhibit the inflammatory response [50,51]. A previous study demonstrated that the hydroethanolic extract of *Moringa oleifera* flowers has anti-inflammatory potentials by preventing the phosphorylation of NF-κB in RAW 264.7 macrophages stimulated with LPS [52]. Moreover, the study showed that the ethanolic extract of *M. concanensis* relieved pain and had anti-inflammation effects by reducing the synthesis of prostaglandin [53]. Thus, these previous studies support our results that *M. concanensis* had inhibitory effects on LPS-induced inflammatory responses in HaCaT cells. In addition, in DNCB-challenged BALB/c mice, we found that the mice treated with *M. concanensis* had significantly improved skin lesions, ear thickness and TEWL. Note that, as shown in Figure 6E, the levels of TEWL were reduced from Day 15. It is thought that the TEWL levels were decreased by hair regrowth. Moreover, our results show that the mice treated with *M. concanensis* had a reduced spleen index, IgE levels in plasma, epidermal thickness, mast cell infiltration and NLRP3 inflammasome expression in lesional ear tissue. Therefore, our data indicate the anti-atopic properties of *M. concanensis* in DNCB-challenged BALB/c mice.

Accumulating evidence suggests that inflammasomes are associated with the inflammatory response in AD [54,55]. In particular, NLRP3 inflammasome-mediated IL-1β plays a critical role in the pathological process of inflammation-mediated skin diseases [56]. Thus, inhibition of NLRP3 inflammasome-dependent IL-1β could regulate the pathogenesis of AD. The present study revealed that *M. concanensis* inhibited the priming signal of the NLRP3 inflammasome by restraining NF-κB phosphorylation. This evidence supports the notion that *M. concanensis* inhibits the priming and activating signals of the NLRP3 inflammasome. Moreover, several studies reported that extracts of *M. concanensis* had antioxidative properties [57,58]. In this study, various chemical compounds, including quercetin and quinic acid, were detected using a UPLC-QTOF analysis of *M. concanensis* (Table 1). Some studies reported that quercetin and quinic acid derivatives had anti-inflammatory properties in an animal model of colitis and microglia, respectively [59,60,61]. Notably, previous studies reported that quercetin elicited an inhibitory effect of NLRP3 inflammasome activation in macrophages and endothelial cells [62,63]. These previous reports implicate that quercetin and quinic acid in *M. concanensis* may contribute to the anti-inflammatory effects of *M. concanensis*. Therefore, we considered that quercetin in *M. concanensis* may alleviate the AD symptoms by reducing the activation of NLRP3 inflammasome-mediated IL-1β. Although inhibitors of the NLRP3 inflammasome have anti-inflammatory properties in mice, evidence of similar effects in human skin diseases is still lacking. Therefore, clinical studies targeting the NLRP3 inflammasome for inflammatory skin diseases are needed.

## 4. Materials and Methods

### 4.1. Animals

BALB/c mice (female, 6 weeks old) were procured from Orient Bio (Seongnam, Korea). All animal experiments were performed with the approval of the Institutional Animal Care and Use Committee of the Laboratory Animal Research Center, Kangwon National University, Korea (KW-211208-5). Each cage contained four mice, which were housed under controlled conditions of 21–25 °C and a 12 h light and dark cycle. The animals were given free access to food and water throughout the experimental period. 

### 4.2. Preparation of an Ethanolic Extract of M. concanensis 

The leaves of *M. concanensis* were collected Coimbatore District, Tamil Nadu, India and the plant sample was authenticated (Letter No. BSI/SRC/5/23/2018/Tech-437) Botanical Survey of India, Southern Regional Centre, Coimbatore, Tamil Nadu, India. After washing with distilled water, the leaves were dried under light-shielding conditions. Then, 100 g of dried *M. concanensis* leaves (MC) were mixed with 1 L of 70% ethanol for extraction, twice for 2 h by using an ultrasonic bath. After the extraction, the filtrate was evaporated using a rotary vacuum evaporator. Then, the semisolid residue was lyophilized to produce the extract with 20%. 

### 4.3. Identification of Phytochemicals in M. concanensis by UPLC-QTOF-MS/MS

The phytochemicals in *M. concanensis* L. were estimated by using ultra-performance liquid chromatography supplied with quadrupole time-of-flight mass spectrometry (UPLC/QTOF-MS/MS) (WATERS XEVO GS-XS QTOF analyzer). Ten milligrams of *M. concanensis* were dissolved in 10 mL of 70% ethanol, and then, 2 μL of *M. concanensis* were injected into a Waters ACQUITY UPLC BEH C18 column (50 × 2.1 mm, 1.7 μm). The flow rate of the column was altered at 0.3 mL/min. The mobile phase contained 0.1% formic acid in water (solvent A), and 0.1% formic acid in acetonitrile (solvent B). The column conditions and the characterization of chemical components were followed according to the method described by Oh et al. [64]. The chemical components in the leaves of *M. concanensis* were identified from the library of traditional Chinese medicine (TCM) using UNIFI 1.8 (Waters, Milford, MA, USA) software and an in-house library.

### 4.4. Materials

Dulbecco’s modified Eagle’s medium (DMEM), Dulbecco’s phosphate-buffered saline (DPBS), DEPC water and penicillin-streptomycin (P/S) were procured from Welgene (Gyeongsan, Korea). Fetal bovine serum (FBS) was provided by Atlas Biologicals (Fort Collins, CO, USA). Griess reagent, lipopolysaccharides from *Escherichia coli* O26:B6 (LPS), 3-(4,5-dimethylthiazol-2-yl)-2,5-diphenyl tetrazolium bromide (MTT), dimethyl sulfoxide (DMSO), sodium nitrite, skim milk powder, 1-chloro-2,4-dinitrobenzene (DNCB) and dexamethasone were obtained from Sigma Chemical Co. (St. Louis, MO, USA). RNAiso Plus was purchased from Takara Bio Inc. (Kusatsu, Japan). Chloroform, 2-propyl alcohol, olive oil and acetone were purchased from Daejung (Seongnam, Korea). iNOS, COX-2, IL-6, IL-1β and β-actin oligonucleotide coupled primers were synthesized by Integrated DNA Technologies (Coralville, IA, USA). An enzyme-linked immunosorbent assay (ELISA) kit for prostaglandin E_2_ (PGE_2_) was obtained from R&D Systems (Minneapolis, MN, USA), and an ELISA kit for interleukin-6 (IL-6) was obtained from Abcam (Cambridge, UK). An ELISA kit for interleukin-1β (IL-1β) was obtained from Invitrogen (Carlsbad, CA, USA). TransScript^®^ All-in-One First-Strand cDNA Synthesis SuperMix for qPCR (One-Step gDNA Removal) was purchased from TransGen Biotech Co. (Beijing, China). PowerSYBR^®^ Green PCR Master Mix from Applied Biosystems was purchased from Thermo Fisher Scientific (Rockford, IL, USA). P38, c-Jun N-terminal kinase (JNK), extracellular signal-regulated kinase (ERK), P65, phosphorylated P38 (p-P38), phosphorylated JNK (p-JNK), phosphorylated ERK (p-ERK) and phosphorylated P65 (p-P65) antibodies were procured from Cell Signaling Technology (Danvers, MA, USA). All other reagents and materials were of the highest quality available.

### 4.5. Cell Culture

The human epidermal keratinocyte cells (HaCaT) were provided by Professor Ok-Hwan Lee from the Food Chemistry Laboratory at Kangwon National University. HaCaT cells (2 × 10^5^) were cultured in DMEM supplemented with 10% FBS in a 5% CO_2_ incubator at 37 °C. LPS was used to stimulate the HaCaT cells at the concentration of 1 μg/mL for 1 h or 24 h.

### 4.6. Cell Viability

The viability of HaCaT cells was determined using an MTT assay. For this purpose, the cells were pretreated with *M. concanensis* for 24 h and incubated with MTT solution at 5 mg/mL for 4 h to form formazan crystals. After the incubation, the supernatant in each well was replaced with 100 μL of DMSO and isopropyl alcohol (1:1). The absorbance was measured at 540 nm on SpectraMax microplate reader (Molecular Devices, Sunnyvale, CA, USA). 

### 4.7. Nitric Oxide Production

HaCaT cells were pretreated with different concentrations of *M. concanensis* (10, 30, 100 and 300 μg/mL) for 1 h and then treated with LPS (1 μg/mL) for 24 h. The production of nitric oxide (NO) was assessed by measuring the nitrite accumulation in the culture medium. The level of nitrite in the medium was measured by Griess reagent. Briefly, 100 μL of supernatant and Griess reagent were mixed and incubated for 10 min. The absorbance was measured at 540 nm on SpectraMax microplate reader (Molecular Devices, Sunnyvale, CA, USA). The level of nitrite in the culture supernatant of LPS-induced HaCaT cells was calculated using a sodium nitrite standard curve. 

### 4.8. RNA Extraction and Real Time Quantitative Polymerase Chain Reaction (RT-qPCR)

The mRNA expression of iNOS, COX-2, TNF-α, IL-1β, IL-6, NLRP3 and ASC was measured using RT-qPCR. The total RNA was extracted using RNAiso PLUS (Takara, Otsu, Japan). Complementary DNA (cDNA) was synthesized from 1 μg of total RNA using All-in-One FirstStrand cDNA Synthesis SuperMix previously described by Ko et al. [65]. The synthesized cDNAs were used as a template for RT-qPCR using a QuantStudio 3 (Applied Biosystems, Foster City, CA, USA) system with POWER SYBR Green PCR master mix and gene-specific primers (Table 2). A dissociation curve analysis of iNOS, COX-2, IL-1β, IL-6, NLRP3, ASC and β-actin demonstrated a single peak. The expression levels of the target genes were quantified by duplicating measurements and normalized with the 2^−ΔΔCT^ method relative to the control β-actin. The PCR analyses were performed under the following conditions: 40 cycles of 95 °C for 15 s; 57 °C for 20 s, and 72 °C for 40 s.

### 4.9. PGE_2_, TNF-α, IL-1β, IL-6 and IgE Assays

The expression of PGE_2_, TNF-α, IL-1β and IL-6 in the culture supernatant was measured using ELISA kits (R&D Systems, Minneapolis, MN, USA). The cells were pretreated with *M. concanensis* at various concentrations (10, 30, 100 and 300 μg/mL) for 1 h and stimulated with LPS (1 μg/mL) for 24 h. The expression of IgE in plasma was also measured using an ELISA kit (R&D Systems, Minneapolis, MN, USA) according to the manufacturer’s protocol.

### 4.10. Western Blot Analysis

The cell cultures were washed with Dulbecco’s phosphate-buffered saline (DPBS) on ice and proteins were isolated from the cells using lysis buffer (Jubiotech, Daejeon, Korea) with a protease phosphatase inhibitor cocktail (Thermo Fisher Scientific, Rockford, IL, USA). The total cellular proteins were quantified using a Bradford assay, and protein (20 μg/well protein) was loaded onto 10% SDS-PAGE gels and then transferred to PVDF membranes [66]. The membranes were blocked with 5% skimmed milk for 2 h and then incubated with primary antibodies against iNOS (Cell Signaling Technology, 1:1000), COX-2 (Cell Signaling Technology, 1:1000), NLRP3 (Cell Signaling Technology, 1:500), ASC (Cell Signaling Technology, 1:500), Caspase-1 (Cell Signaling Technology, 1:500), p-p65 (Cell Signaling Technology, 1:1000), p-JNK (Cell Signaling Technology, 1:1000), p-ERK (Cell Signaling Technology, 1:1000), p-p38 (Cell Signaling Technology, 1:1000), p-c-fos (Cell Signaling Technology, 1:1000), p65 (Cell Signaling Technology, 1:1000), JNK (Cell Signaling Technology, 1:1000), ERK (Cell Signaling Technology, 1:1000), p38 (Cell Signaling Technology, 1:1000), c-fos (Cell Signaling Technology, 1:500), or GAPDH (Cell Signaling Technology, 1:500) at 4 °C overnight. After washing, the membranes were incubated for 2 h with a secondary antibody. The protein bands were detected using enhanced chemiluminescence (ECL) (General Electric, Boston, MA, USA). Subsequently, the proteins were visualized using a LAS-500 mini-imager and quantified with ImageJ software (version 1.51j8).

### 4.11. 2,4-Dinitrochlorobenzene (DNCB)-Induced Atopic Dermatitis Mice

**Figure 9:**
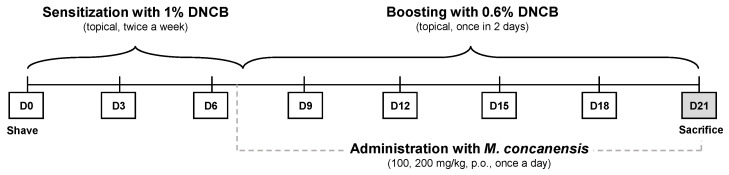
Diagram of the experimental procedures.

BALB/c mice were topically sensitized with 200 μL of 1% DNCB diluted in a mixture of acetone and olive oil (3:1), on shaved dorsal skin and ears twice a week. The mice were divided into 5 groups (n = 8/group) as follows: an untreated group (Normal), an only DNCB-sensitized group (DNCB), a group receiving oral administration of 100 mg/kg *M. concanensis* (MC 100), a group receiving oral administration of 200 mg/kg *M. concanensis* (MC 200) and a group receiving the administration of 1 mg/kg dexamethasone (DEXA). Seven days later, the mice were stimulated with 0.6% DNCB on the dorsal skin (200 μL) and the right ear (20 μL for every 2 days). Mice with DNCB-induced AD-like skin lesions were orally treated with *M. concanensis* (100 and 200 mg/kg) and dexamethasone (1 mg/kg) every day (Figure 9).

### 4.12. Measurement of Clinical Symptoms and Histological Changes

The severity of the skin lesions in DNCB-induced AD was estimated according to the SCORAD index, which is scored from 0 (none) to 3 (severe) based on erythema, pruritus/dry skin, edema and excoriation [67]. The thickness of the ear was measured using a Digimatic micrometer (Mitutoyo, Kawasaki, Japan). GPSKIN Barrier Pro (GPpower, Hanam, Korea) was used to measure transepithelial water loss (TEWL) in the dorsal skin using the GPSKIN Research program [68]. Changes in the clinical symptoms, such as body weight, ear thickness and TEWL, in the AD mice were measured every three days. To evaluate the histological examination, the dorsal skin tissues were punched using a 5 mm biopsy punch, fixed in 10% formalin solution and embedded in paraffin [69]. Each section slice of paraffin-embedded skin tissue was stained with hematoxylin and eosin (H&E) and toluidine blue (TB). The histological changes were examined by light microscopy (Olympus, Tokyo, Japan). The epidermal thickness was observed using H&E staining at 100× magnification. The infiltration of mast cells was analyzed with TB staining and the slices were examined in four randomly selected sections. 

### 4.13. Statistical Analysis

The statistical analyses were performed using GraphPad Prism Version 8.0 (GraphPad, La Jolla, CA, USA). All data are expressed as the mean ± S.E.M. The data were analyzed by a one-way analysis of variance (ANOVA), followed by a Student–Newman–Keuls test for multiple comparisons. *p* < 0.05 was considered a significant statistical value.

## 5. Conclusions

In conclusion, our results show that *M. concanensis* inhibited the formation of the NLRP3 inflammasome through JNK/AP-1/NF-κB signaling in HaCaT keratinocytes. Furthermore, we suggest that *M. concanensis* attenuated DNCB-induced AD-like symptoms in BALB/c mice by inhibiting IL-1β mediated by the NLRP3 inflammasome. Therefore, *M. concanensis* has therapeutic properties in chronic inflammatory skin diseases, mainly AD.

## Figures and Tables

**Table 1:** Phytochemical profiling of *M. concanensis* Leaves.

RT (min)	Tentative Identification	Formula	m/z [M-H]^−^	Mass Error (ppm)	Response	Fragmentation (m/z)
0.46	Maltose	C_12_H_22_O_11_	341.1090	−1.3	1,038,656	179.0561
0.47	Quinic acid	C_7_H_12_O_6_	191.0559	−1.3	1,983,085	85.0301
0.50	Coumaroylquinic acid	C_16_H_18_O_8_	337.0918	−3.3	111,547	191.0561
0.62	Malic acid	C_4_H_6_O_5_	133.0137	−3.9	27,811	89.0246, 114.9995
0.89	4-O-Caffeoylquinic acid	C_16_H_18_O_9_	353.0874	−1.2	180,161	135.0452, 173.0445, 191.0562
0.98	Esculin	C_15_H_16_O_9_	339.0715	−2	2322	177.0191
1.03	tryptophan	C_11_H_12_N_2_O_2_	203.0823	−1.5	31,577	116.0510, 142.0665
1.13	Coumaric acid	C_9_H_8_O_3_	163.0402	1.1	44,900	119.0504
1.15	Coumaroylquinic acid	C_16_H_18_O_8_	337.0926	−0.9	505,720	163.0402, 173.0454, 191.0562
1.24	Hydroxybenzoic acid	C_7_H_6_O_3_	137.0244	−0.2	14,703	93.0351
1.25	Coumaric acid	C_9_H_8_O_3_	163.0403	1.1	7702	93.0351, 119.0505
1.30	4-Feruloylquinic acid	C_17_H_20_O_9_	367.1030	−1.3	25,889	134.0376, 193.0509
1.44	Apigenin 6,8 C-dihexose	C_27_H_30_O_15_	593.1513	0.3	626,091	353.0671, 473.1010
1.49	Coumaroylquinic acid	C_16_H_18_O_8_	337.0926	−0.8	131,771	173.0455, 191.0562
1.55	Coumaroylquinic acid	C_16_H_18_O_8_	337.0926	−0.8	110,283	163.0402, 173.0454
1.73	Orientin	C_21_H_20_O_11_	447.0934	0.2	2271	327.052
1.78	isopentyl β-primeveroside	C_16_H_30_O_10_	381.1766	0.6	1,128,403	249.135
2.09	Quercetin hydroxymethylglutaroyl glycoside	C_27_H_28_O_16_	607.1307	0.4	51,494	300.0287
2.15	Vitexin	C_21_H_20_O_10_	431.0983	−0.3	135,245	283.0617, 311.0566, 341.0674
2.31	Kaempferol-3-O-β-D-glucopyranoside	C_21_H_20_O_11_	447.0936	0.8	3530	285.041
2.45	Nicotiflorin	C_27_H_30_O_15_	593.1520	1.3	576,122	285.0409
2.54	Isorhamnetin 3-O-rutinoside	C_28_H_32_O_16_	623.1620	0.4	209,986	300.0279, 315.0514
2.74	Isorhamnetin 3-glucoside	C_22_H_22_O_12_	477.1038	−0.2	7248	299.0205, 314.0435
2.94	Azelaic acid	C_9_H_16_O_4_	187.0975	−0.5	78,016	125.0972
3.07	Unknown	C_23_H_34_O_13_	517.1930	0.7	447,712	-
3.71	Quercetin	C_15_H_10_O_7_	301.0354	0.2	6398	151.0032
4.15	Unknown	C_21_H_36_O_10_	447.2234	0.2	493,377	-
4.88	Kaempferide	C_16_H_12_O_6_	299.0557	−1.5	3863	284.0326
8.63	(E,E)-9-Oxooctadeca-10,12-dienoic acid	C_18_H_30_O_3_	293.2123	0.2	367,072	-
8.57	Unknown	C_30_H_54_ N_2_O_19_	745.3250	0.3	30,373	-
8.74	(E,E)-9-Oxooctadeca-10,12-dienoic acid	C_18_H_30_O_3_	293.2121	−0.5	436,590	-
9.45	Coronaric acid	C_18_H_32_O_3_	295.2280	0.6	119,446	-
10.00	Ricinoleic acid	C_18_H_34_O_3_	297.2434	−0.5	9993	-
10.27	Unknown	C_34_H_40_ N_2_O_5_	555.285	−2.6	1,277,907	-
11.54	Ursolic Acid	C_30_H_48_O_3_	455.3528	−0.5	144,205	-
11.83	Linolenic acid	C_18_H_30_O_2_	277.2174	0.2	108,147	-
11.96	Pentadecanal	C_15_H_30_O	[M + COOH]^−^ 271.2277	−0.6	60,182	-
13.09	n-Heptadecanal	C_17_H_34_O	[M + COOH]^−^ 299.2591	−0.3	73,029	-
13.70	Oleic acid	C_18_H_34_O2	281.2486	0.1	45,678	-

**Table 2:** The list of primer sequences used in the RT-qPCR analyses.

Target Gene		Primer Sequence
*iNOS*	F	5′-CAT GCT ACT GGA GGT GGG TG-3′
	R	5′-CAT TGA TCT CCG TGA CAG CC-3′
*COX-2*	F	5′-TGC TGT ACA AGC AGT GGC AA-3′
	R	5′-GCA GCC ATT TCC TTC TCT CC-3′
*TNF-α*	F	5′-AGC ACA GAA AGC ATG ATC CG-3′
	R	5′-CTG ATG AGA GGG AGG CCA TT-3′
*IL-1β*	F	5′-ACCT GCT GGT GTG TGA CGT T-3′
	R	5′-TCG TTG CTT GGT TCT CCT TG-3′
*IL-6*	F	5′-GAG GAT ACC ACT CCC AAC AGA CC-3′
	R	5′-AAG TGC ATC ATC GTT GTT CAT ACA-3′
*NLRP3*	F	5′-GCGTGTTGTCAGGATCTCGCATTGG-3′
	R	5′-GTGTCTCCAAGGGCATTGCTTCGTAG-3′
*ASC*	F	5′-ACAGAAGTGGACGGAGTGCT-3′
	R	5′-CTCCAGGTCCATCACCAAGT-3′
*β-actin*	F	5′-ATC ACT ATT GGC AAC GAG CG-3′
	R	5′-TCA GCA ATG CCT GGG TAC AT-3′

## Data Availability

All data are included in the article.
